# Risk factors for mortality after nocardiosis in Southern France (2014-2024)

**DOI:** 10.1016/j.ijregi.2026.100961

**Published:** 2026-07-23

**Authors:** Faustine Girardet, Benjamin Coiffard, Sébastien Cortaredona, Sophie Edouard, Frédérique Gouriet, Jean-Christophe Lagier, Nadim Cassir

**Affiliations:** 1 IHU-Méditerranée Infection, Marseille, France; 2 Aix Marseille Univ, Department of Respiratory Medicine and Lung Transplantation, AP-HM, Hôpital Nord, Marseille, France; 3 Aix-Marseille Univ, IRD, SSA, MINES, Marseille, France; 4 Aix Marseille Univ, SSA, RITMES, Marseille, France; 5 Aix-Marseille Université, AP-HM, MEPHI, Marseille, France

**Keywords:** Nocardiosis, Solid organ transplants, Lymphopenia, Mortality, Nocardia farcinica, Antibiotic susceptibility testing

## Abstract

**Objectives:**

To analyze the risk factors associated with 1-year overall mortality in patients with nocardiosis.

**Methods:**

This retrospective multicenter study was conducted across four university hospitals within the same health care network over 11 years (2014-2024) in Marseille, France. Age-adjusted exact multivariable logistic regression was performed to identify factors associated with 1-year overall mortality.

**Results:**

Of the 78 patients with a positive *Nocardia* spp. sample, 52 (66.7%) had a confirmed infection. Of these patients, 53.8% (28/52) had advanced forms, 34.6% (18/52) had disseminated forms, and 65.4% (34/52) had localized forms. The 1-year mortality rate was 21.2% (11/52), reaching 37.5% (six of 16) in the transplant subgroup and 32.1% (nine of 28) in patients with an advanced form. Severe lymphopenia (<0.5 G/l) was independently associated with 1-year mortality (odds ratio 5.70; 95% confidence interval 1.15-35.23). The main species identified was *Nocardia abscessus* (16/48, 33%), followed by *Nocardia farcinica* (9/48, 19%). Neither species was associated with an increased risk of 1-year mortality. All isolates except one were susceptible to imipenem (98%). Ninety-seven percent were susceptible to amikacin, 96.5% to linezolid, and 79% to trimethoprim-sulfamethoxazole. Mortality did not differ according to the adequacy of initial antibiotic therapy, based on antibiotic susceptibility testing or reference charts.

**Conclusions:**

Solid organ transplantation and advanced disease were associated with higher crude 1-year mortality rates. In the age-adjusted multivariable analysis, severe lymphopenia (<0.5 G/l) was the only factor independently associated with 1-year mortality.

## Highlights

• Solid organ transplant recipients were at increased risk of dying within 1 year.
• Patients with an advanced form of nocardiosis were at increased risk of dying within 1 year.
• Antibiotic adequacy based on the antibiogram or reference charts had no impact.
• Severe lymphopenia was independently associated with 1-year overall mortality.

## Introduction

*Nocardia* species are aerobic, filamentous, Gram-positive bacilli commonly found in soil, water, and decaying vegetation [1]. Non-disseminated cutaneous nocardiosis typically results from direct inoculation following skin trauma or skin barrier disruption and primarily affects immunocompetent individuals [2]. In contrast, pulmonary or disseminated nocardiosis (defined as infection involving two non-contiguous organs or bacteremia) is caused by *Nocardia* colonizing the airways. These forms of the disease primarily affect individuals with chronic lung diseases, such as bronchiectasis or chronic obstructive pulmonary disease, as well as those with immunocompromising conditions [2]. The most common cause of an immunocompromised state among patients with nocardiosis is iatrogenic immunosuppression resulting from transplantation, anti-neoplastic therapy, or treatment for autoimmune disease. Primary immunodeficiencies, notably chronic granulomatous disease, are also associated with nocardiosis [3]. The number of people in these at-risk populations is growing [4].

*Nocardia* species exhibit variable antimicrobial susceptibility, which can be partially predicted by species identification. Identifying the species allows timely adjustment of empirical antibiotic therapy, thereby minimizing the risk of ineffective treatment before susceptibility results become available [2]. When susceptibility testing is not possible, clinicians refer to published literature tables [[5], [6], [7], [8], [9]].

Mortality rates and risk factors for mortality associated with nocardiosis vary widely depending on the characteristics of the study populations and the duration of follow-up [[10], [11], [12], [13]]. A recent multicenter cohort study of nocardiosis in Japan found that a Charlson Comorbidity Index >5 and disseminated nocardiosis were associated with 1-year mortality [12]. However, two retrospective cohort studies found that disseminated infection was not associated with mortality in their multivariable analyses [14,15]. In contrast, advanced infection (defined as dissemination, pleural involvement, cavitary pulmonary infection, or central nervous system [CNS] involvement), a higher CCI, and infection caused by *N. farcinica* were all independently associated with mortality [14]. These discrepancies suggest that prognostic factors remain incompletely characterized and may vary across populations. Furthermore, most studies have focused on clinical presentation and comorbidities, whereas easily available biological markers reflecting host immune status have been less extensively investigated [12,14,16]. Contemporary European data outside transplant settings also remain scarce. Finally, the prognostic value of severe lymphopenia has not been specifically investigated in previous cohorts. Therefore, we aimed to identify factors associated with 1-year overall mortality in patients with nocardiosis managed in Southern France.

## Methods

### Study design and setting

We conducted an 11-year retrospective multicenter cohort study at four university hospitals in Marseille, collectively known as “Assistance Publique - Hôpitaux de Marseille” (AP-HM). These hospitals account for approximately 125,000 annual hospitalizations and 320 annual solid organ transplants. Using the database of the microbiology laboratory at the IHU-Méditerranée Infection in Marseille, we selected all patients with a positive clinical sample for *Nocardia* spp. between January 2014 and December 2024. We then included patients presenting with clinical signs, symptoms, or radiographic findings consistent with *Nocardia* infection. After selecting cases for inclusion, we extracted the data manually from the electronic medical records. The following variables were recorded for each patient: demographic data (age and sex), underlying conditions (e.g., chronic bronchitis, cystic fibrosis, autoimmune disease, solid organ transplant, solid cancer, blood cancer, hematopoietic stem cell transplant, and HIV infection), immunosuppressive treatment (e.g., high-dose corticosteroids [≥10 mg/day for ≥21 days], calcineurin inhibitors, chemotherapy, anti-CD20 monoclonal antibodies, and methotrexate), trimethoprim-sulfamethoxazole (TMP-SMX) prophylaxis at the time of infection (e.g., dosage of 800 mg of trimethoprim/160 mg of sulfamethoxazole three times a week or 400 mg of trimethoprim/80 mg of sulfamethoxazole once daily), symptoms experienced, disease extent, type of clinical sample, lymphocyte count, type of imaging, microbiological testing, *Nocardia* species, antimicrobial susceptibility testing, treatment (antibiotics or surgical procedure), duration of treatment, and vital status (“dead” or “alive”) at 1 year.

### Definitions

We considered a patient to have nocardiosis if they had a positive *Nocardia* spp. sample and exhibited signs, symptoms, or imaging findings indicative of the disease. We defined disseminated disease as infection involving two non-contiguous organs or bacteremia [[17], [18], [19], [20]]. We defined advanced infection as disseminated infection, CNS infection, or pleural or cavitary forms [14]. Unlike Yetmar et al. [14]*,* isolated CNS involvement was not classified as disseminated disease but was included in the definition of advanced disease because of its recognized severity and potential prognostic implications. We defined *Nocardia* colonization as a *Nocardia* spp.-positive sample without signs or symptoms of infection, a *Nocardia* spp.-positive sample with signs or symptoms explained by another cause, or a *Nocardia* spp.-positive sample with signs or symptoms that did not progress with treatment [14,21,22]. We considered solid cancer an underlying condition in patients with progressive disease (clinical or radiographic evidence of progression) and/or those undergoing active treatment.

### Microbiology

*Nocardia* isolates recovered in culture were identified at the species level using matrix-assisted laser desorption ionization–time of flight mass spectrometry (MALDI-TOF MS) [23]. In selected cases, 16S ribosomal RNA polymerase chain reaction (PCR) [24] was performed on sterile-site specimens, skin biopsies, or at the clinician’s request. A specific *N. farcinica* real-time PCR assay was also available and was performed only at the clinician’s request [25]. Antimicrobial susceptibility testing was interpreted according to the recommendations applicable during the study period. When broth microdilution was available, minimum inhibitory concentrations were interpreted according to Clinical and Laboratory Standards Institute recommendations for *Nocardia* species [26]. For isolates tested by E-test or disk diffusion, interpretation was performed according to the recommendations of the European Committee on Antimicrobial Susceptibility Testing and the French Society of Microbiology [27]. For probabilistic antibiotic therapy, published susceptibility data for the relevant *Nocardia* species were used [[5], [6], [7], [8], [9]].

### Statistical analysis

The primary objective of this study was to identify risk factors associated with 1-year overall mortality. Bivariate analyses were conducted using Fisher’s exact test for categorical variables and the Wilcoxon–Mann–Whitney test for continuous variables. For the primary objective, variables with a *P*-value <0.20 in the bivariate analyses were considered candidates for inclusion in a multivariable logistic regression model. As there were only 11 deaths in the cohort, the number of variables included in the multivariable model was intentionally limited to minimize the risk of overfitting and unreliable estimates. Age was included in the model because of its recognized clinical importance as a potential confounding factor, and severe lymphopenia was chosen as the second explanatory variable. Exact logistic regression was used to obtain valid estimates despite the small number of events. We also performed receiver operating characteristic curve analyses to determine the optimal cutoff value for lymphocyte count using Youden index (*Y* = sensitivity + specificity − 1). All statistical analyses were performed using SAS software, version 9.4 (SAS Institute, Cary, NC). Statistical significance was set at *P* < 0.05.

### Ethical aspects

This study was approved and registered in the AP-HM Health Data Access Portal under registration number PADS24-314 and approved by the AP-HM Ethics and Scientific Committee under the approval number CSE24-53.

## Results

### Characteristics of the patients

Over the 11-year period, we identified 78 patients with positive culture or PCR test results for *Nocardia* species. Seven of these patients were considered colonized and excluded (7/78, 8.9%). Clinical records could not be retrieved for 18 patients (23.1%), and one patient was excluded because of their opposition to the use of their health data for research purposes. The final study population therefore consisted of 52 patients with confirmed nocardiosis. The median age was 57.5 years, and the median Charlson Comorbidity Index score was 3. The two most prevalent underlying diseases were solid organ transplantation (16/52, 30.8%) and chronic respiratory disease (17/52, 32.7%). Among the patients with chronic respiratory disease, seven had chronic obstructive pulmonary disease, five had cystic fibrosis, and five had bronchiectasis. This was followed by autoimmune and inflammatory diseases (6/52, 11.5%), including sarcoidosis, Horton disease, and ankylosing spondylitis. Two patients were found to have anti-granulocyte macrophage colony-stimulating factor (GM-CSF) antibodies following immunological investigations. In both patients, *Nocardia* species were isolated from pulmonary specimens, and one patient also had concomitant CNS involvement. Only one patient (one of 52, 1.9%) had a primary immunodeficiency, namely X-linked agammaglobulinemia (Bruton). Five patients (five of 52, 9.6%) had a solid tumor, and four (4/52, 7.7%) had hematologic malignancies, all of whom had lymphoma. Two patients had cirrhosis, and one had HIV with a CD4 count below 500/mm³. Among solid organ transplant recipients, the most common type of transplant was kidney (eight of 16, 50%), followed by lung (five of 16, 31.2%), heart (two of 16, 12.5%), and combined heart–lung (one of 16, 6.3%). Supplementary Table S1 shows the characteristics of patients according to their transplant status Data were available for all patients except two, for whom “lymphocyte count” data were missing. Solid organ transplant recipients had a statistically higher incidence of severe lymphopenia (lymphocytes <0.5 G/l or <500/mm³) (60.0% vs 17.1%, *P* = 0.006) than the other patients. They were also more likely to receive immunosuppressive treatment (100% vs 19.4%, *P* < 0.001) than the rest of the population. No statistically significant differences were observed regarding the extent of nocardiosis, patient age or infection with *N. farcinica*. The median time from transplantation to the onset of nocardiosis was 44 months (27 months for kidney transplantation, 16.2 months for lung transplantation, and 123.6 months for heart transplantation). All transplant patients were on immunosuppressants. Eleven patients (68.6%) were on a combination of an antimetabolite and a calcineurin inhibitor, and six patients (37.5%) were on high-dose corticosteroids. Only one patient received prophylactic treatment with TMP-SMX at a dosage of 400 mg trimethoprim and 80 mg sulfamethoxazole once daily. This patient was a 60-year-old kidney transplant recipient who developed nocardiosis within the first year after transplantation. The isolated species was *Nocardia abscessus*, which was susceptible to TMP-SMX.

Thirty-four patients presented with a localized form of the disease. Among these patients, 19 (19/34, 55.9%) had an isolated pulmonary form, including one patient with pleural involvement. Nine patients (nine of 34, 26.5%) had an isolated cutaneous form, five patients (five of 34, 14.7%) had an isolated neurological form, and one patient (one of 34, 2.9%) had an isolated osteoarticular form. Disseminated forms accounted for 34.6% (18/52) of cases. Supplementary Figure S1 shows the different organ associations of disseminated forms. The most frequent association was between the CNS and lungs (eight of 18, 44.4%). Bloodstream infections occurred in 16.7% (three of 18) of patients, none of whom had a central venous catheter. Twenty-eight patients (28/52, 53.8%) had advanced disease. Of these patients, 18 had disseminated forms (defined as involvement of two non-contiguous organs) (18/52, 34.6%), eight had a cavitary lesion (eight of 52, 15.4%), two had a pleural infection (two of 52, 3.8%); one had a pleural infection associated with a cavitary lesion (one of 52, 1.9%), and five had an isolated CNS form (five of 52, 9.6%). In cases involving the CNS, the most common presentation was a central neurological deficit (14/18, 77.8%), followed by confusion (nine of 18, 50.0%), headaches (seven of 18, 38.9%), and coma (one of 18, 5.6%). Cerebral abscesses were the predominant manifestation, except in one patient who presented with meningitis and another who presented with the nodular form. Among the three patients with CNS involvement but no neurological symptoms, cerebral lesions were identified during the evaluation of disseminated nocardiosis. In one patient, CNS involvement was confirmed post-mortem by histopathological examination of brain tissue. In the remaining two patients, brain magnetic resonance imaging revealed cerebral lesions, whereas microbiological confirmation was obtained from extracerebral sites. Among the 34 cases of pulmonary form (3 pleural forms and 31 parenchymatous forms), 11 patients (11/34, 32.3%) showed no respiratory symptoms. The remaining 23 patients exhibited various symptoms. Sixteen patients (16/23, 69.6%) experienced dyspnea, 14 (14/23, 60.9%) had a cough, and 13 (13/23, 56.5%) produced sputum. All patients received antibiotic therapy. A surgical procedure was performed in 44.2% (23/52) of cases, primarily for neurological forms (13/18, 72.2%). The most used first-line antibiotic was TMP-SMX (32/52, 61.5%). The carbapenem + TMP-SMX combination accounted for 41.7% (15/36) of combination therapies. Among survivors at 1 year, the median duration of antibiotic treatment was six months, with 36.6% (15/41) treated for fewer than 120 days.

### Species and antimicrobial susceptibility

**Figure 1:**
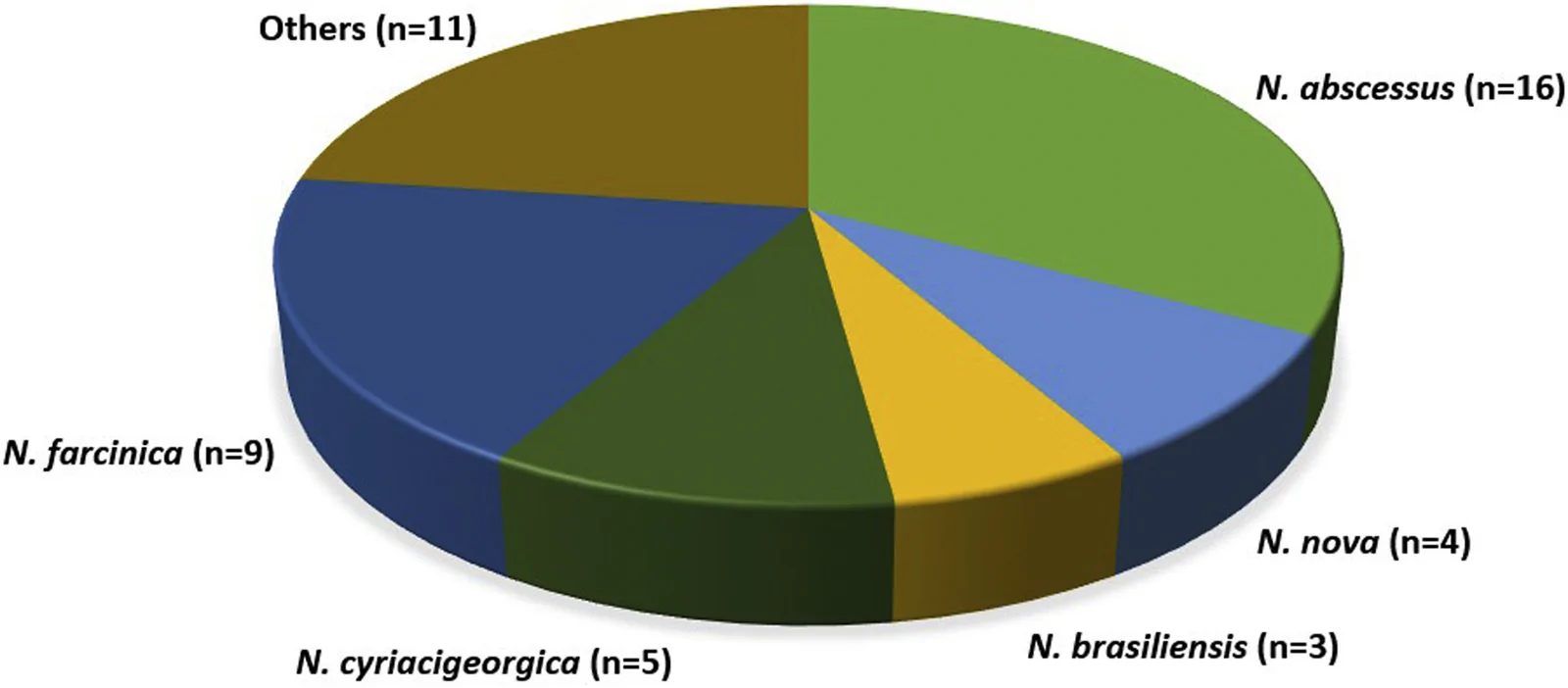
Distribution of the *Nocardia* species identified. * Others include *Nocardia wallacei* (n = 2), *Nocardia paucivorans* (n = 2), *Nocardia asteroides* (n = 2), *Nocardia barduliensis* (n = 1), *Nocardia beijingensis* (n = 1), *Nocardia otitidiscaviarum* (n = 1), *Nocardia puris* (n = 1), *Nocardia tadekensis* (n = 1).

Culture and PCR identified the *Nocardia* species in 92.3% (48/52) of cases: 6.2% (three of 48) by PCR alone and 93.8% (45/48) by culture. The most common species were *N. abscessus* (16/48; 33.3%) and *N. farcinica* (nine of 48; 18.8%), including among the solid organ transplant population. Figure 1 shows the distribution of the different *Nocardia* species identified. Supplementary Table S2 summarizes the different antimicrobial susceptibility profiles according to *Nocardia* species. High variability in antibiotic susceptibility was observed among species. All species except one (*Nocardia otitidiscaviarum*) were susceptible to imipenem. Overall susceptibility rates were 98.0% for imipenem, 97.0% for amikacin, 96.5% for linezolid, and 79.1% for TMP-SMX. Among the 18 patients with disseminated nocardiosis, the same *Nocardia* species was identified in all sampled anatomical sites in 17 cases. One patient had two different *Nocardia* species isolated from separate specimens.

### Risk factors for overall mortality at 1 year

In our study, the 1-year all-cause mortality rate was 21.2% (11/52). Nine of the 11 patients who died had advanced disease, including eight with disseminated forms and one with isolated CNS form. Table 1 shows the characteristics of the study population according to their vital status as alive or dead at 1 year. Data were collected for all patients except two, for whom lymphocyte count data were missing. Patients who died within the first year were significantly more likely to have profound lymphopenia (lymphocytes <0.5 G/l) (63.6% vs 20.5%, *P* = 0.010) and advanced infection (81.8% vs 46.3%, *P* = 0.046) than those who survived. The initial antibiotic regimen was considered appropriate in 31 cases (59.6%) according to both isolate-specific antibiotic susceptibility testing (AST) and published susceptibility data for the identified *Nocardia* species. In the remaining 21 cases (40.4%), treatment adequacy depended on whether it was assessed using AST or published susceptibility charts. Mortality did not differ according to antibiotic adequacy based on the AST (nine of 42 (21.4%) vs two of 10 (20.0%), *P* = 1.000) or reference charts (eight of 37 (21.6%) vs three of 15 (20.0%), *P* = 1.000). After adjusting for age, severe lymphopenia remained statistically associated with an increased likelihood of dying within 1 year (odds ratio 5.70; 95% confidence interval 1.15-35.23; *P* = 0.031; see Table 2). The optimal lymphocyte cutoff to predict all-cause mortality, as determined by the Youden index, was 520/mm³ (0.52 G/l), with a sensitivity of 72.7%, a specificity of 79.5%, and an area under the curve of 0.79 (95% confidence interval 0.61-0.96) (Supplementary Figure S2).

**Table 1:** Risk factors for 1-year mortality.

	Alive (n = 41)		Dead (n = 11)		*P*-value^a^	Total (n = 52)	
	n	%	n	%		n	%
**Sex - men**	21	51.2	7	63.6	0.516	28	53.8
**Age - mean (SD) Q1-median-Q3**	53.2 (21.8)	42-57-70	59.7 (16.8)	45-60-78	0.392	54.6 (20.9)	44-58-72
**Solid organ transplant**	10	24.4	6	54.6	**0.073**	16	30.8
**Charlson Comorbidity Index**							
Mean (SD) Q1-median-Q3	3.5 (2.6)	2-3-5	5.1 (3.3)	2-5-8	0.120	3.9 (2.8)	2-3-5
≥5	11	26.8	6	54.6	0.145	17	32.7
**Immunosuppressant use**	16	39.0	7	63.6	0.182	23	44.2
**Localized form**	31	75.6	3	27.3	**0.005**	34	65.4
**Advanced form**	19	46.3	9	81.8	**0.046**	28	53.8
**Skin/soft tissue**	12	29.3	4	36.4	0.720	16	30.8
**CNS**	12	29.3	6	54.6	0.159	18	34.6
**Lungs**	27	65.9	6	54.6	0.503	33	63.5
**Lymphocyte count (nmiss=2)**							
Mean (SD) Q1-median-Q3	1512.3 (1091.2)	580-1400-2000	704.5 (936.2)	220-400-860	0.006	1334.6 (1103.0)	400-990-1900
<500	8	20.5	7	63.6	**0.010**	15	30.0
***Nocardia farcinica***	8	19.5	1	9.1	0.664	9	17.3
***Nocardia abscessus***	11	26.8	4	36.4	0.709	15	28.9
**Antibiotics conformity with the AST**	33	80.5	9	81.8	1.000	42	80.8
**Antibiotics conformity with the reference charts**	29	70.7	8	72.7	1.000	37	71.2

Univariate analysis (n = 52).

AST, antibiotic susceptibility testing; CNS, central nervous system.

a Fisher’s exact test for qualitative variables; Wilcoxon–Mann–Whitney test for quantitative variables. (Lymphocytes are presented as /mm³).

**Table 2:** Risk factors for 1-year mortality. Multivariable age-adjusted exact logistic regression (*n* = 50).

Effect	OR 95% CI^a^	*P*
**Age**^b^	1.01 0.97-1.06	0.689
**Lymphocytes <500 (Ref. Lymphocytes ≥500)**	5.70 1.15-35.23	**0.031**

a Adjusted exact odds ratios with 95% confidence intervals.

b Age was forced into the model. Other covariates were selected using a stepwise procedure among variables associated with 1-year mortality in univariate analysis (*P* < 0.20, Table 1).

## Discussion

We present the findings of a multicenter study conducted across four university hospitals within the same healthcare network that investigated 52 cases of nocardiosis, focusing particularly on factors associated with mortality within the first year. Our study found an overall 1-year mortality rate of 21.2%. After adjusting for age, profound lymphopenia (defined as less than 0.5 G/l or <500/mm³) was independently associated with increased 1-year mortality. These findings highlight the role of profound lymphopenia as a marker of impaired host immune response and poor prognosis in nocardiosis. Our findings are consistent with those of Soueges et al. [16], who identified lymphopenia as an independent risk factor for disseminated nocardiosis in a multicenter French cohort. Together, these results suggest that impaired cellular immunity may contribute to both disease dissemination and adverse clinical outcomes. Although advanced disease and solid organ transplantation were associated with higher 1-year mortality rates, these factors were not significantly and independently associated with mortality in the multivariable analysis. This differs slightly from the results of a study by Yetmar et al. [14], in which advanced infection was a better predictor of 1-year mortality than disseminated infection in a multivariable analysis, whereas immunocompromised status was not a significant predictor. However, our study found no statistically significant difference regarding Charlson Comorbidity Index or *N. farcinica*, contradicting the findings of Yetmar et al. [14] and Takamatsu et al. [12]. In their work, the severity of comorbidities and the *N. farcinica* species were associated with a poor prognosis. This discrepancy may reflect differences in sample size, population characteristics, or statistical power. Furthermore, first-line antibiotic therapy, whether in accordance with susceptibility charts or the subsequently received AST, did not appear to influence 1-year mortality. These findings suggest that host-related factors could play a more central role in determining prognosis than early antibiotic selection alone.

The mortality rate was 37.5% in the solid organ transplant group, higher than that reported by Coussement et al. [28] and Yetmar et al. [14]. Several factors may explain this difference, including differences in patient characteristics, degree of immunosuppression, and the small sample size of our transplant subgroup. In the European cohort by Lebeaux et al. [13], time from transplantation to nocardiosis was associated with mortality in univariable analysis but did not remain significant after multivariable adjustment. Other factors identified as prognostic in these studies include fungal infection in the six months prior to diagnosis and advanced donor age. These factors were not studied in our cohort and could also explain the difference in mortality rates.

We identified three patients with CNS involvement and 11 patients with pulmonary involvement who presented without symptoms related to their respective organ involvement. This is particularly true among immunocompromised patients. For instance, in a study by Vega Brizneda et al. [15], only 50% of the cohort with CNS involvement presented with neurological symptoms. Although advanced infection was not independently associated with mortality in our adjusted model, these data should encourage the systematic use of brain and thoracoabdominal imaging in immunocompromised patients [14].

In our study, we were able to identify the species in 92.3% of cases. However, we were unable to investigate whether the absence of species-level detection was associated with mortality, as reported by Lu et al. [29].

AST revealed that all strains except one (*Nocardia otitidiscaviarum*) were sensitive to imipenem (98%), supporting its use as a first-line antibiotic agent. Furthermore, 96.5% of strains in the cohort were susceptible to linezolid, 97% to amikacin, and 79% to TMP-SMX. These results are consistent with those published by Lebeaux et al. [5] and Yetmar et al. [14], with the exception of TMP-SMX, for which 94.6% of strains were susceptible. However, the observed resistance rate should be interpreted cautiously and may partly reflect technical limitations of susceptibility testing rather than true resistance. For instance, *in vitro* TMP-SMX resistance in *Nocardia* spp. may have been overestimated because of methodological issues [30,31].

Thirty-one percent of our cohort consisted of solid organ transplant recipients, with kidney transplantation being the most common type of transplant (50% of cases). Our data are consistent with recent European studies indicating that solid organ transplantation is the primary underlying condition associated with invasive nocardiosis, accounting for 25%-40% of cases [13]. Only one patient in our transplant subgroup was receiving prophylactic treatment with TMP-SMX at the time of diagnosis (dosage: trimethoprim 400 mg and sulfamethoxazole 80 mg once daily). The median time between transplantation and infection was 44 months, exceeding the recommended duration of *Pneumocystis jirovecii* prophylaxis [28]. Recent data suggest that TMP-SMX prophylaxis is partially protective against nocardiosis in both solid organ transplant recipients and allogeneic hematopoietic stem cell transplant recipients, although breakthrough infections may still occur. These findings raise the possibility of extending prophylaxis or implementing personalized immune monitoring strategies in persistently lymphopenic patients [3,32,33].

In our cohort, two patients with no pre-existing conditions at the time of diagnosis underwent immunological testing that revealed the presence of anti-GM-CSF autoantibodies. This finding aligns with the current literature, which emphasizes the importance of immunological testing in patients who appear healthy but present with nocardiosis [31].

Our study has several limitations. First, it was a retrospective study, which introduces inherent biases. Second, although conducted across four university hospitals, the study was restricted to a single healthcare network in Southern France, which may limit the generalizability of our findings to other geographic areas. Third, the small sample size may limited our ability to detect an association between advanced infection and mortality. The small number of deaths also limited the complexity of the multivariable model, which may have reduced our ability to detect independent associations with other clinically relevant variables. Additionally, differences in the definition of disseminated disease across studies may prevent direct comparisons of prognostic factors. In contrast to Yetmar et al. [14], we did not classify isolated CNS involvement as disseminated disease in our study but instead included it in the advanced disease category. However, reclassifying the five patients with isolated CNS nocardiosis as having disseminated disease did not significantly change the observed association between disseminated disease and mortality (one patient died within 1 year).

## Conclusion

In this multicenter cohort of patients with nocardiosis, severe lymphopenia (<0.5 G/l) was the only factor independently associated with 1-year mortality after age adjustment. These findings suggest that profound lymphopenia may serve as a simple and readily available marker of poor prognosis in nocardiosis. Systematic assessment of lymphocyte counts could help identify high-risk patients who may benefit from closer monitoring and optimized management strategies.

## CRediT authorship contribution statement

**Faustine Girardet:** Data curation, Formal analysis, Writing – original draft. **Benjamin Coiffard:** Data curation, Validation. **Sébastien Cortaredona:** Formal analysis, Methodology, Validation. **Sophie Edouard:** Data curation, Validation. **Frédérique Gouriet:** Data curation, Validation. **Jean-Christophe Lagier:** Resources, Validation. **Nadim Cassir:** Conceptualization, Data curation, Formal analysis, Project administration, Writing – review & editing.

## Declaration of competing interest

All authors have no conflicts of interest to report.

## Appendix Supplementary materials

Image, application 1
