# Supplementary material for: Risk factors for mortality after nocardiosis in Southern France (2014-2024)
Source: IJID Reg. 2026 Jul 23;20:100961. doi: 10.1016/j.ijregi.2026.100961 (PMC13505432; doi:10.1016/j.ijregi.2026.100961)

Supplementary appendix

Table S1. Characteristics of patients according to their solid organ transplant status (n=52).

	NonSOT (n=36)		SOT (n=16)		p-value[†]	All (n=52)	
	n	%	n	%		n	%
**Sex - Men**	21	58.3	7	43.8	0.378	28	53.9
**Age – Mean (std) Q1-Median-Q3**	53.7 (23.3)	42-57-73	56.6 (14.3)	48-60-67	0.945	54.6 (20.9)	44-58-72
**Charlson Comorbidity Index**							
Mean (std) Q1-Median-Q3	3.6 (3.0)	1-3-6	4.4 (2.0)	3-4-5	0.116	3.9 (2.8)	2-3-5
≥5	11	30.6	6	37.5	0.751	17	32.7
**Immunosuppressant use**	7	19.4	16	100.0	**<.001**	23	44.2
**Localized form**	26	72,2	8	50.0	0.206	34	65.4
**Advanced form**	18	50.0	10	62.5	0.549	28	53.9
**Skin/soft tissue**	9	25.0	7	43.8	0.206	16	30.8
**Central nervous system**	13	36.1	5	31.3	1.000	18	34.6
**Lungs**	23	63.9	10	62.5	1.000	33	63.5
**Lymphocytes (nmiss=2)**							
Mean (std) Q1-Median-Q3	1479.1 (1114.1)	580-1400-1900	997.3 (1034.3)	260-410-2000	0.091	1334.6 (1103.0)	400-990-1900
< 500	6	17.1	9	60.0	**0.006**	15	30.0
Nocardia farcinica	4	11.1	5	31.3	0.113	9	17.3
Nocardia abscessus	11	30.6	4	25.0	0.752	15	28.9
**Antibiotics conformity with the AST**	27	75.0	15	93.8	0.147	42	80.8
**Antibiotics conformity with the reference charts**	25	69.4	12	75.0	0.752	37	71.2
**Death at one year**	5	13.9	6	37.5	0.073	11	21.2

†: Fisher's exact test for qualitative variables; Wilcoxon–Mann–Whitney test for quantitative variables.

SOT=solid organ transplant; AST=antibiotic susceptibility testing

Figure S1. Organ association in disseminated forms

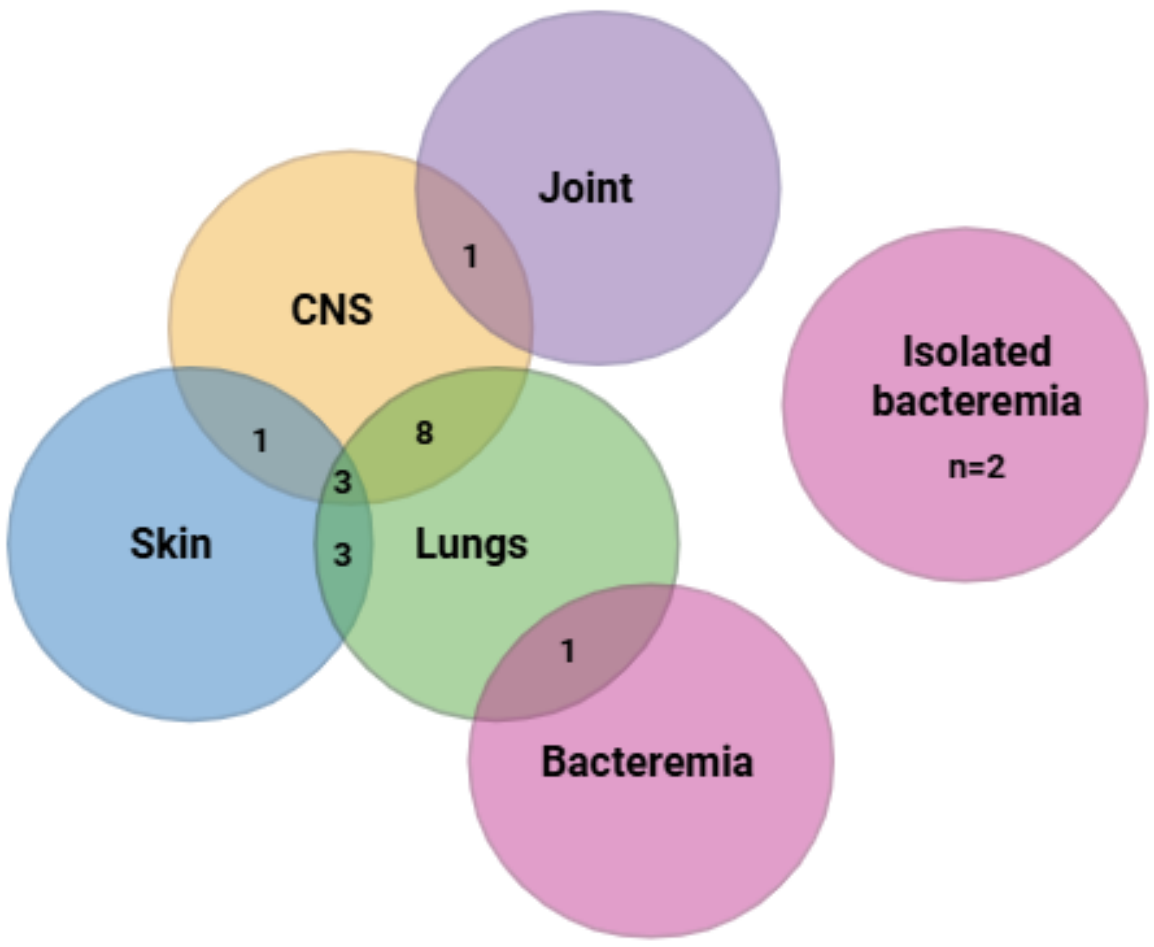

(CNS + lungs: 8; skin + lungs: 3; Lung + bacteriemia: 1; skin + CNS + lungs: 3; Isolated bacteremia: 2; CNS + skin: 1; CNS + joint: 1). CNS=central nervous system.

Table S2. Antimicrobial susceptibility profile of the different Nocardia isolates.

	Amoxicillin - clavulanic acid	Ceftriaxone	Imipenem	Meropenem	Ciprofloxacin	Linezolid	TMP-SMX	Doxycycline	Amikacin	Gentamicin	Vancomycin
Nocardia abscessus (n=14)	92% (11/12)	75% (3/4)	100% (14/14)	100% (8/8)	0% (0/7)	90% (9/10)	93% (13/14)	87,5% (7/8)	100% (9/9)	Non tested	60% (6/10)
Nocardia farcinica (n=8)	100% (8/8)	0% (0/3)	100% (8/8)	60% (3/5)	0% (0/4)	100% (4/4)	86% (6/7)	50% (2/4)	100% (8/8)	0% (0/2)	25% (2/8)
Nocardia cyriacigeorgica (n=5)	25% (1/4)	100% (3/3)	100% (5/5)	100% (1/1)	0% (0/1)	100% (1/1)	50% (2/4)	100% (1/1)	100% (4/4)	75% (2/3)	50% (2/4)
Nocardia nova (n=4)	50% (2/4)	25% (1/4)	100% (4/4)	100% (1/1)	0% (0/2)	100% (3/3)	100% (4/4)	100% (2/2)	100% (1/1)	0% (0/1)	45% (3/4)
Nocardia brasiliensis (n=3)	100% 2/2	0% (0/1)	100% (3/3)	100% (1/1)	100% (2/2)	100% (2/2)	50% (1/2)	Non tested	100% (2/2)	Non tested	0% (0/1)
Nocardia asteroides (n=2)	50% (1/2)	100% (2/2)	100% (2/2)	50% (1/2)	Non tested	100% (1/1)	50% (1/2)	100% (1/1)	100% (1/1)	Non tested	100% (2/2)
Nocardia wallacei (n=2)	100% (1/1)	Non tested	100% (2/2)	100% (1/1)	100% (2/2)	100% (1/1)	100% (1/1)	100% (2/2)	100% (2/2)	0% (0/1)	0% (0/2)
Nocardia paucivorans (N=2)	100% (2/2)	100% (2/2)	100% (2/2)	Non tested	100% (1/1)	100% (1/1)	50% (1/2)	100% (2/2)	100% (1/1)	Non tested	100% (2/2)
Nocardia barduliensis (n=1)	R	S	S	S	Non tested	S	S	Non tested	S	Non tested	Non tested
Nocardia beijingensis (n=1)	S	S	S	Non tested	R	S	S	S	Non tested	Non tested	S
Nocardia puris (n=1)	S	S	S	S	Non tested	S	S	S	R	Non tested	S
Nocardia otitidiscaviarum	R	Non tested	R	R	Non tested	Non tested	S	Non tested	S	Non tested	R
All strains* (n=48)	76% (31/41)	71% (15/21)	98% (47/48)	83% (20/24)	28.5% (6/21)	96,5% (28/29)	79% (34/43)	85% (23/27)	97% (32/33)	28% (2/7)	56.5% (22/39)

*Both identified (n=44) and unidentified species (n=4) strains for which an antibiogram could be performed. R= resistant; S= susceptible.

Figure S2. Determination of the optimal cut-off for lymphocyte level to predict all-cause mortality: ROC curve analysis

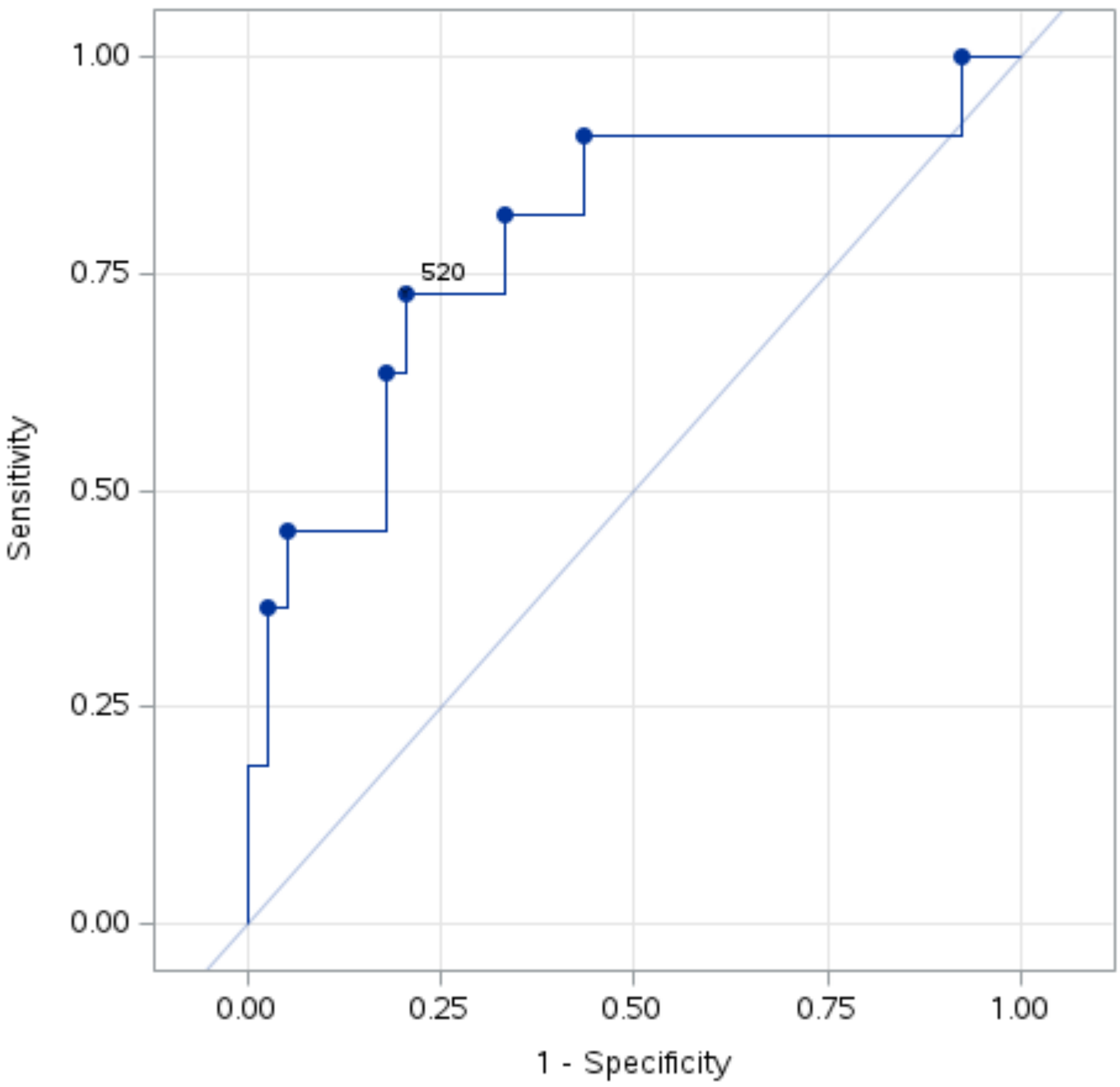

Supplement: Supplementary file 1 [file mmc1.pdf]
